# Efficacy of Mobile Phone Short Message Service (SMS) Reminders on Malaria Treatment Adherence and Day 3 Post-Treatment Reviews (SMS-RES-MAL) in Kenya: A Study Protocol

**DOI:** 10.4172/2167-0870.1000217

**Published:** 2015-03-23

**Authors:** Ambrose O Talisuna, Dejan Zurovac, Sophie Githinji, Amos Oburu, Josephine Malinga, Andrew Nyandigisi, Caroline OH Jones, Robert W Snow

**Affiliations:** 1 Department of Public Health Research, KEMRI-Welcome Trust Research Program, Kenya; 2 Centre for Tropical Medicine, Nuffield Department of Clinical Medicine, University of Oxford, UK; 3 Malaria Control Unit, Ministry of Health, Nairobi, Kenya

**Keywords:** Efficacy, Mobile phone, Short message service (SMS), Malaria treatment, Adherence, Day 3 post treatment reviews, Randomized controlled trial

## Abstract

**Background:**

Mobile phone short messaging services (SMS) have been investigated in health information reporting, provider performance, drug and diagnostic stock management and patient adherence to treatment for chronic diseases. However, their potential role in improving patients’ adherence to malaria treatment and day 3 post treatment reviews remains unclear.

**Methods/Design:**

A “proof of concept” open label randomised controlled trial will be conducted at four sites in Western Kenya. Principal research questions are: 1) Can mobile phone SMS reminders improve patient adherence to malaria treatment? 2) Can mobile phone SMS reminders improve day 3 post treatment reviews? Eligible caregivers (n=1000 per arm) of children under five years old with uncomplicated malaria will be randomly assigned (one to one) to: a) the current standard of care (provider counselling and health education); and b) the current standard of care plus SMS reminders. Within each arm, caregivers will be further randomized to three different categories. In categories 1 and 2, 300 caregivers per arm per category will be visited at home on day 1 and 2 of follow up respectively, to measure appropriate timing and adherence of the second Artemether-Lumefantrine (AL) dose and doses 3 and 4. Further, caregivers in categories 1 and 2 will be required to come to the health facility for the day 3 post treatment reviews. Finally, in category 3, 400 caregivers per arm will be visited at home on day 3 to measure adherence for the full AL course. Each category will be visited at home only once to avoid biases in the measures of adherence as a result of home consultations. Primary outcomes will be adherence to the full AL course (category 3), as well as, the proportion of patients reporting back for day 3 post treatment reviews (categories 1 and 2). The primary analysis will be intention-to-treat. Costs of the intervention will be measured over the period of the intervention, and a cost-effectiveness ratio will be estimated.

**Discussion:**

If successful, evidence from this trial could improve malaria treatment adherence and offer pragmatic approaches for antimalarial drug resistance surveillance and risk mitigation in Africa.

**Current Controlled Trials:**

ISRCTN39512726

## Background

Artemisinin resistant *Plasmodium falciparum* malaria has emerged in south East Asia (SEA), with possible foci in Western Cambodia, Western Thailand, Myanmar and possibly beyond [1–3]. This poses a major global public health threat, with the greatest potential effects in sub-Saharan Africa where the malaria burden is greatest and systems for resistance surveillance and risk mitigation are weakest. A public health disaster is imminent unless mitigation, early warning and early detection measures are urgently set up in Africa. The main phenotypic trait associated with artemisinin resistance is a substantial delay in parasite clearance, so far observed in SEA but not yet unequivocally confirmed in Africa [4]. Despite calls for the reactivation of drug resistance surveillance networks in Africa [5,6]; coordinated and proactive efforts to address the threat of artemisinin resistance are limited. A recent case report of severe malaria that manifested after 4 days in a patient who returned to Vietnam from Angola, where he had lived for 3 years, and was not responsive to intravenous artesunate plus clindamycin or oral dihydroartemisinin-piperaquine needs further investigation [7].

Early detection of emerging Artemisinin resistance in Africa is likely to require wide coverage surveillance, beyond the traditional sentinel surveillance. Consequently, robust collection of the proportion of patients who remain parasite positive at day, if feasible, could offer highly sensitive and pragmatic metrics for tracking emerging Artemisinin resistance [8,9]. Such metrics could provide early evidence of a congruence of factors in “hotspots”, which could be designated, drug resistance surveillance sites; be targeted for investigative clinical trials with 6-8 hourly parasite density sampling; or could be targeted for molecular surveillance, as and when molecular markers for artemisinin resistance become well established and validated [6,10]. However, in Africa, routinely conducting day 3 post treatment reviews to estimate the proportion of patients with persistent patent parasitaemia after 72 hours is often not carried out. Consequently, innovative ways of promoting this practice are urgently needed.

Poor patient adherence to treatment and drug misuse are among some of the drivers of resistance [11]. Poor adherence is likely to decrease cure rates and to favour the emergence of resistance because parasites are exposed to sub therapeutic drug concentrations. Evidence from systematic reviews suggests variable outcomes of adherence to malaria treatment, ranging from sub-optimal (less than 50%) to very high (90-100%) [12–15]. The varying adherence estimates are probably due to factors related to patient characteristics and the nature of their consultation with the provider or methodological variations such as the interaction between the research team and patients before and during treatment. A recent systematic review has suggested that future studies of adherence to malaria treatment could benefit from an awareness of the impact of study procedures on adherence outcomes, and has proposed the identification of improved measurement methods less dependent on self-reporting [13]. Presently, the most widely used methods for adherence measurement are conducted after completion of a full dose of Artemether-Lumefanthrine (AL). However, these methods rarely consider the timing of dosing and depend on self-reporting which has inherent recall bias resulting in unreliable measurements of adherence to AL dosing schedules. AL is presently the most widely used artemisinin combination therapy (ACT) in rural Africa, but AL has a relatively complex dosing scheme, requiring twice a day drug administration with the second dose spaced 8 hours after the first dose. From literature review, only one study has attempted to measure the timing of individual AL doses [16]. There is therefore a need to investigate better approaches for measuring and improving adherence to malaria treatment and this research aims to provide the much needed evidence base.

Mobile phone short messaging services (SMS) could improve the delivery of health services in Africa and their role has been investigated in health information reporting, provider performance, drug and diagnostic stock management and patient adherence to treatment for chronic diseases such as HIV/AIDs [17–25]. However, the efficacy of SMS reminders in improving patients’ adherence and day 3 post treatment reviews for acute diseases such as malaria has not been widely investigated. At the time of writing this protocol, no peer reviewed studies had been published that have investigated the impact of SMS reminders sent to caregiver’s mobile phones on malaria treatment adherence and day 3 post treatment reviews. In Kenya, the coverage of mobile phones has increased in the recent past and many districts have universal mobile network coverage [26,27]. The efficacy of mobile phone SMS reminders in improving malaria treatment adherence and day 3 post treatment reviews in children with uncomplicated malaria in Kenya will be investigated in an endeavour to improve malaria treatment adherence as well as investigate novel approaches for early warning and early detection of emerging Artemisinin resistance in Africa.

## Methods/Design

### Overall study design

The research is being conducted in three phases. Phase I, was an assessment of the feasibility, patterns of use and acceptability of using mobile phone SMS reminders for malaria treatment adherence and day 3 post treatment review. Thereafter, through an iterative process involving multi-disciplinary teams, the research team developed; pilot tested and finalized the intervention. Phase II, which commenced in June 2014, is a randomized controlled trial (RCT) to assess the efficacy of the SMS intervention. Finally, in phase III, qualitative research to describe the factors contributing to the success or failure of the intervention and cost-effectiveness analysis of the intervention with respect to the primary outcomes–adherence and day 3 post treatment reviews will be undertaken.

### Trial sites

**Figure 1:**
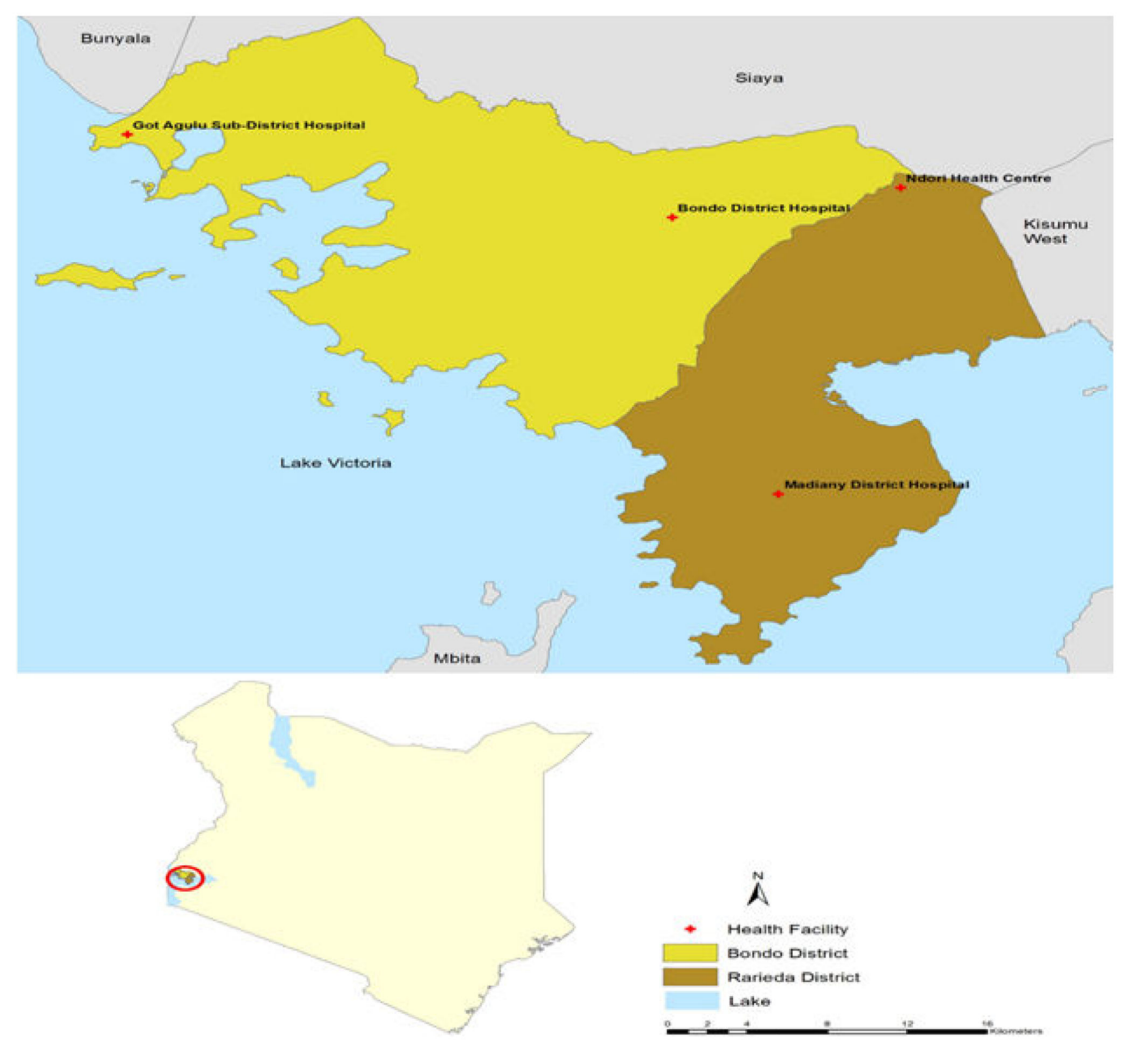
All trial sites.

The research is being conducted at four sites in Kenya. Two sites (Bondo and Got Agulu hospitals) are located in Bondo district, while the other two sites (Ndori health centre and Madiany hospital) are located in Rarieda district (Figure 1). All trial sites are government owned public health facilities. Bondo District hospital is a referral facility and the only one located in an urban area. According to the 2009 census, Bondo and Rarieda districts have a combined population of 29,080 with a density of 997 persons per km^2^. The population is predominantly of Luo ethnicity and earns its living through subsistence farming and fishing. The area is largely rural with urban settlement limited to one town, Bondo. These two districts have high malaria transmission (parasite prevalence among children 2-10 years above 40%) [28]. More detailed descriptions of the study sites are published elsewhere [29].

### Description of the study phases

**Phase 1–Development of the intervention:** The intervention is based on a one-way automated distribution of SMS reminders for AL adherence and day 3 post-treatment reviews sent to caregivers’ personal mobile phones or phones that they have access to in the household. The primary objective of phase 1 of the research was the development, refinement and finalisation of the intervention. The detailed descriptions of the intervention development process and the qualitative research conducted will be published elsewhere [Githinji et al., manuscript in preparation]. In brief the intervention development process was conducted through a six step process, namely:

**Step 1:** Despite the high coverage of the network signal and mobile phone access from nationally representative surveys [26,27] facility-based exit surveys were conducted at the study sites to validate these national statistics and gather additional information from caregivers pertinent to the development and delivery of the SMS intervention. The details of the feasibility assessment have been published elsewhere [29]. Briefly, out of 400 interviewed caregivers, nearly all (99.8%) reported access to a mobile signal at home. Ninety three percent (93%) had access to a mobile phone within their household while 73.8% possessed a personal phone. Among caregivers with mobile phone access, 93.6% used the phone to receive text-messages. The willingness to receive text-message reminders was nearly universal (99.7%) with 41.7% of caregivers preferring texts in English, 32.3% in Kiswahili and 26.1% in Dholuo language [29].

**Step 2:** In this step, the various modalities of the intervention delivery were considered, including: the number, content, length, frequency and languages to be used for text messages. An intervention development workshop was conducted, involving clinicians and caregivers from the areas neighbouring the study sites, malaria epidemiologists, social scientists, mobile health programmers, and members of the district health management teams.

**Step 3:** The understanding of the messages developed was pre-tested using focus group discussions (FGDs). FGDs were conducted among caregivers from the study sites to ensure that the messages were clearly understood by the target group. Twenty caregivers of sick children from the study health facilities were invited to participate in the FGDs during which they received SMS text messages in Dholuo, English or Kiswahili based on their language preference. Members of the groups discussed the meaning and understanding of the messages. Following the testing, the messages were amended as required.

**Step 4:** A computerized SMS distribution system was developed by mobile phone programmers to ensure the automated SMS distribution to individual recipients upon their registration into the system. The system is a web-based application platform optimized for data collection and reporting. It is composed of: (1) an SMS data collection tool which uses a built-in SMS gateway to receive and send structured SMS messages according to the language option preferred by the caregiver, which are then stored in a local database and (2) an analytics tool that enables visualization and analysis of data sent via SMS to help real time tracking of the operational progress of the trial. Upon registration, the system creates a schedule of reminder messages to be sent at specific timings and generates log reports of all messages scheduled and those already sent.

**Step 5:** Pilot testing of the automated distribution system and the selected messages was conducted, during a complete delivery of the intervention to 40 healthy recipients including all study investigators, and 20 malaria caregivers at health facilities not involved in the RCT. All malaria caregivers receiving the messages were visited by a social scientist to solicit their views and experiences of the pilot intervention.

**Step 6:** Finally, based on the results of the pilot test, the content and modalities of the text-message distribution was finalized before being deployed in the RCT. The English version of the text-messaging content with timing of SMS distribution is presented in Table 1.

### Phase 2 – The randomized controlled trial

**Trial design:** Caregivers of eligible children with uncomplicated malaria will be randomly assigned (one to one) to two different arms: 1) the current standard of care based on provider counselling and health education alone, and 2) the current standard of care plus SMS reminders. Within each arm participants will be further randomly assigned to 3 different categories for the measurement of adherence to the different doses of AL. In the first category, 600 caregivers (300 per arm) will be visited at home on day 1 of follow up to measure adherence and timing of the second AL dose; in the second category, another 600 caregivers (300 per arm) will be visited at home on day 2 to measure adherence and timing of AL doses, 3 and 4, while, in the third category, a final 800 caregivers (400 per arm) will be reviewed at home on day 3 after they have completed the full treatment course to measure adherence for the full course of AL. All caregivers in each category will be visited at home only once to avoid biases in the subsequent measures of adherence as a result of the consultations at home. Caregivers in category 1 and 2 will be required to come to the health facility for the day 3 post treatment reviews, while those in category 3 will be visited at home on day 3.

**Figure 2:**
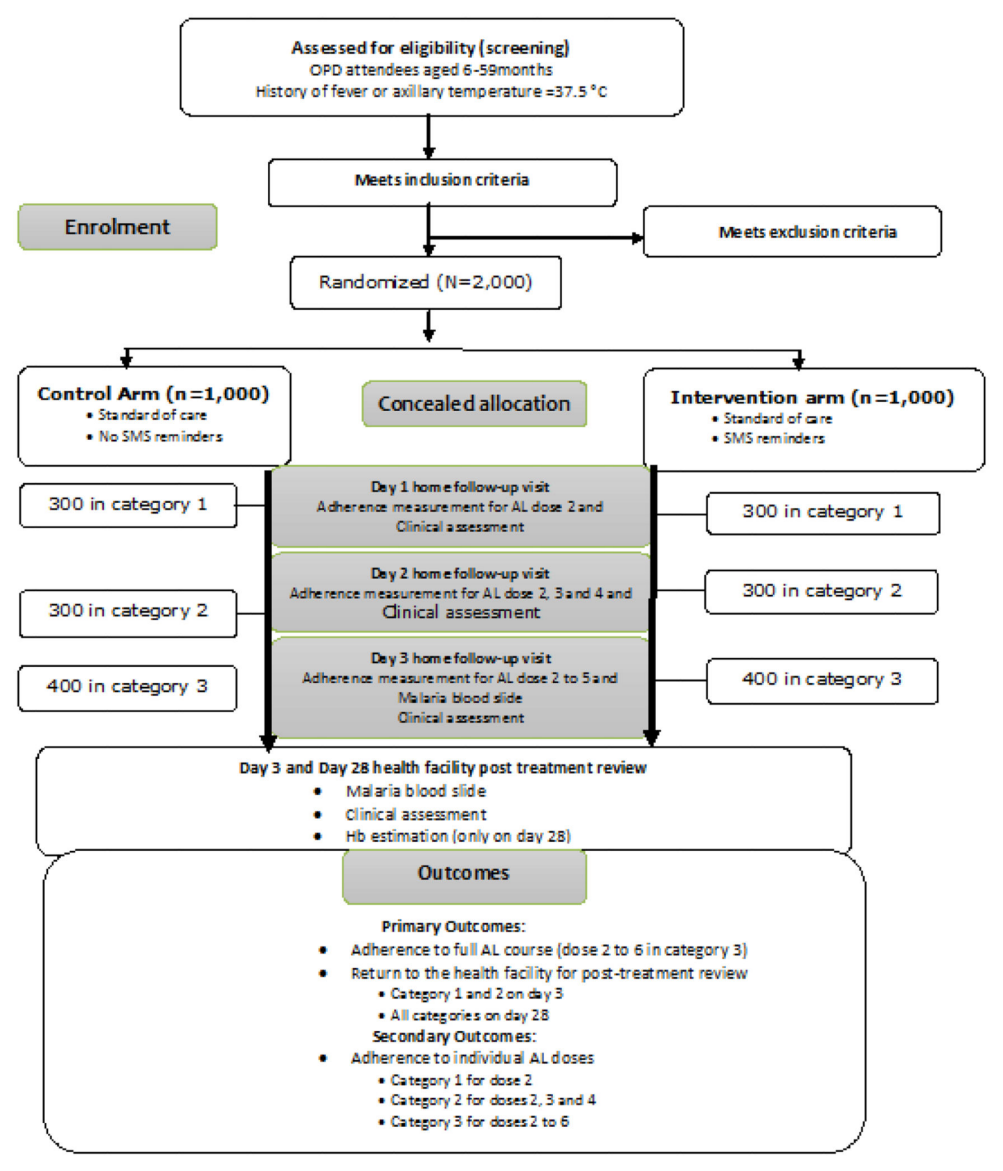
Algoritham.

AL will be administered according to weight bands: 5-14 kg, 1 tablet twice a day for three days, 15-24 kg, 2 tablets twice a day for three days and will be directly observed for the first dose, with the remaining 5 doses being provided for administration at home. All caregivers will receive standard instructions about how to administer AL at home and when to return to the facility for scheduled visits or immediately if unwell. At first dose administration, the telephone numbers of all eligible caregivers will be registered into the automated system by the randomizing nurse who will also observe directly supervised therapy of the first dose. The registration will record the time when the first dose is administered. A procedure for guiding the correct registration of the random number that will act as a unique identifier has been put in place (Figure 2).

In the intervention arm the registration generates a programme that automates SMS reminders from a central server, timed to eight hours after the first AL dose for the second dose and then every morning and evening (8 am and 8 pm) until the full AL course is completed. To limit the number of follow up SMS messages being received at very late hours of the night, patient recruitment will be stopped by 2 pm. Caregivers in category 1 and 2 in the intervention arm will in addition to the SMS dose reminders also receive an SMS reminder for the day 3 post treatment reviews at the health facility the evening before and on the morning of the appointment. Since caregivers in category 3 (intervention and control) will be reviewed at home on day, they will not receive SMS reminders for the day 3 post treatment reviews. Weekly SMS reminders (day 7, 14 and 21) prompting caregivers to visit the health facility if their child is unwell will be sent to participants in the intervention arm. If such reminders result in a visit to the health facility, such a visit will be considered an un-scheduled visit. Finally, participants in the intervention arm will be sent final SMS reminders on the evening before and on the morning of the day 28 post treatment review appointment. Patients who do not return for post treatment review (intervention and control arms) will not be actively followed up.

### Allocation of participants to trial groups

In order to ensure concealment of the intervention allocation to the trial staff, the randomization to the two study arms and subsequently to the 3 adherence measurement categories per arm was generated by an offsite statistician. Intervention allocation is concealed from the study staff reviewing the patients for treatment outcomes until evaluation is completed. However, such concealment could not be implemented for those conducting the home visits since they also collect data on SMS exposure, but we believe this lack of concealment will not affect the adherence outcome because the home visit will be conducted after the AL dose has been administered. Since caregivers will be visited at home only once, we will not measure adherence of the subsequent doses of AL in category 1 and 2. We designed the study to measure adherence to the full course of AL only in category 3 when all doses should have been completed to enable comparison with previous studies.

### Patient recruitment and potential problems with compliance

The districts we have selected have very high malaria transmission intensity. In many malaria clinical trials recruitment rates of 5-7 caregivers per day has been demonstrated. Based on mobile phone ownership data, 50-65 % of the population is likely to own a mobile phone. So we anticipate recruiting 2-3 caregivers per day per site. Patients attending the outpatient department will be screened for eligibility criteria and those eligible will be randomized into the intervention and control arms as well as the adherence measurement categories. While recruitment at the four sites is cumulative we will try to ensure that participants at each of the four sites are as to close to equal as possible (approximately 500 participants per site). Potential challenges related to mobile phone and SMS use such as sharing of personal phones or not charging or switching off phones were determined during phase I and, if widely common, will be reinforced by the health workers to sensitize caregivers about the need to have mobile phones on and charged during the trial period.

### Quality control and limitation of bias

The major source of bias in adherence studies is reliance on participant recall and the possibility of false reporting. We have adopted an innovative approach of measuring adherence on different days during the dosing period, which is likely to reduce recall bias for the doses on days 1 and 2 in category 1 and 2 but not in category 3. The second primary outcome measure (day 3 post treatment review) requires objective assessment and we do not anticipate any biases in its measurement. The trial will be conducted under strict adherence to good clinical practice (GCP) standards.

### Inclusion criteria

Caregivers of male and female children aged <5 years old; body weight eligibility for AL; microscopically confirmed, mono-infection of *Plasmodium falciparum* (parasitaemia ≥ 500/μL to 200,000/μL); history of fever in the last 24 hours or presence of fever (axillary temperature ≥ 37.5°C); network coverage at patients’ household; owning a mobile phone or having shared access to a mobile phone in household; ability to access a mobile phone on a daily basis for the period of follow up; ability to open and read SMS or presence of someone in the household who can open or read an SMS and written informed consent.

### Exclusion criteria

Caregivers not owning a mobile phone; and have no shared access to any phone in the household; patients with severe malaria; danger signs: not able to drink or breast-feed, vomiting (>twice in 24 hours), recent history of convulsions (>1 in 24 h), unconscious state, unable to sit or stand; presence of concomitant illness or any condition which in the judgement of the investigator would place the subject at undue risk or interfere with assessment; severe malnutrition (weight for height<70% of the median NCHS/WHO reference); ongoing prophylaxis with drugs having antimalarial activity such as cotrimoxazole for the prevention of pneumocysti carini pneumonia in children born to HIV+ women; having taken AL in the previous one month and having participated in the current trial.

### Sample size estimation

The sample size estimation was based on the first primary outcome-adherence to the full course of AL treatment. Assuming a malaria treatment adherence of 65% with the current standard of care alone [30], a sample size of 400 participants in category 3 per arm will be able to detect a minimum desirable effect difference of 10% with 80% power at a 0.05 level of significance, assuming a 15% loss to follow up. In order to robustly measure adherence to the individual AL doses, we have included an additional 300 caregivers per arm in each of the first and second categories. This sample size will be sufficient to detect an effect difference of 10% around an estimated adherence of 75% to the individual AL doses, assuming a 10% loss to follow up. For the measurement of the day 3 post-treatment reviews, the combined sample size in categories 1 and 2 (600 per arm), has adequate power (over 90%) to detect a 10% effect difference around a conservative estimate of the proportion returning for day 3 post-treatment review of 45%. Therefore, we will recruit a total 1,000 caregivers for the study.

### Primary outcomes

The first primary outcome will be adherence to a complete AL course (doses 2-6) measured in category 3 only and defined as: a) adherent, if they present with empty blister packs and report taking the medications as recommended (± 1 hour for dose 2 and ± 2 hours for doses 3-6); b) probably adherent if they do not present blister packs but report taking the medications as recommended, c) probably non adherent if they do not present blister packs and do not report taking the medications as recommended, and d) non adherent if they present blister packs with at least one tablet not used. The second primary outcome will be the proportion of patients reporting to the health facility for the day 3 post treatment review for the evaluation of clinical and parasitological cure at day 3 (only category 1 and 2).

### Secondary outcomes

Secondary outcomes will include: adherence and timing for individual AL doses measured in category 1 for AL dose 2 and category 2 for AL doses, 3 and 4, and category 3 for all AL doses. The other secondary outcomes will be loss to follow up at day 3 defined as unable to return to the facility 24 hours after the scheduled appointment.

### Measurement of the efficacy of the SMS intervention on adherence

At each of the time points (day 1, 2 and 3); caregiver reports and pill counts will be used for measuring adherence in the intervention and control arms. While the day 1 and day 2 visits could result into slightly better adherence for doses, 3 and 4 because they are visited at home in the early days of follow up, category 3 will provide closer to real life adherence as these patients will only be visited at home after completion of the treatment course.

Measurement of the efficacy of the SMS intervention on day 3 post treatment reviews

At enrolment, all caregivers will be informed about the importance of a day 3 post treatment review from a clinical and drug resistance surveillance perspective, to confirm a malaria free status at day, or anytime during follow up if unwell for a period of 28 days. The proportion returning for the day 3 post treatment reviews will be determined in the intervention and control arms.

### Laboratory procedures

At enrolment and at all scheduled visits at the health facility or at home (days 0, 3 and 28) and at all unscheduled visits, a blood smear will be taken for the estimation of parasite density. Screening thick blood smears will be stained with 10% locally prepared Giemsa for 10 minutes, while definitive thick and thin blood smears will be stained with 2% commercially prepared Giemsa for 30 minutes. The thick smears will be used to make the diagnosis of malaria, calculate parasite density and look for the presence of gametocytes, while the thin smears will be used for speciation. The number of asexual parasites per 200 WBCs will be counted, and if there are less than 10 parasites per 200 WBCs, the count will be continued until 500 WBCs. Parasite density will be estimated based on the assumption of a standard estimate of 8000 WBCs/UL. A blood smear will be deemed negative, if there are no asexual parasites after counting a hundred high power microscopic fields. In addition, haemoglobin level will be estimated at day 0 and day 28, using a hemocue for the quantitative determination of haemoglobin in blood using a specially designed photometer and micro-cuvettes.

### Study protocol review and ethical approval

This study protocol has been reviewed by six independent peer reviewers nominated by the United Kingdom Medical Research Council (UK-MRC) before the grant was awarded. Further, the study protocol, the informed consent documents and all educational or recruitment material was further reviewed and approved by the Kenya Medical Research Institute ethical review committee and the University of Oxford ethical review board (OXTREC).

### Trial monitoring and oversight

The study will be conducted as multi-site trial with a field coordinator supervising all the study sites. After initiation of the trial, monitoring and supervision of the trial is being conducted to guarantee that all study procedures are being followed in accordance with GCP principles. An independent certified monitor from the East Africa Consortium for Clinical Research (EACCR) will be responsible for monitoring the trial. A Trial Steering Committee (TSC) was constituted before the study commenced to address policy and operational issues related to the study; and protecting the scientific conduct and integrity of the trial. In addition, a Data Monitoring Board (DMB) was constituted, whose main roles are: monitoring the recruitment rates of the trial, revising in blind a significant percentage of the primary outcome data and assessing the quality of the data.

### Trial Data Analysis

Individual patient level pooled analysis of the data from the four sites will be conducted, according to CONSORT standards for reporting RCTs. A random effects model will be used to adjust for site and other covariates. To analyse for effect differences for the primary outcomes, intention to treat (ITT) principles will be used and additional sensitivity and per protocol (PP) analyses will be conducted separately. ITT implies that all randomised patients will be considered in the outcomes as per randomization. Missing data will be imputed as intervention failure (non-adherent or not report for post treatment review), using multiple imputation for missing data not directly related to adherence. The latter is considered the gold standard for handling missing data as it leads to unbiased estimates and standard errors compared to single imputation methods [31]. In the per protocol (PP) analysis, only patients completing the study and have been exposed to the intervention will be included. Category analyses will be conducted to investigate the relationship between adherence and treatment success (clinical and parasitological cure) at day 3. The study is powered adequately, so no interim analyses will be conducted.

### Phase 3 – Post-trial qualitative research and cost-effectiveness analysis

The quantitative study will provide information on the proportion of participants adhering to the treatment regime and the proportion returning for the day 3 post-treatment reviews. However, understanding the factors influencing this behaviour requires a more-in-depth qualitative approach. Following completion of the RCT, in-depth interviews will be conducted with a purposively selected sample of participants to explore their perceptions and experiences of the SMS intervention and to investigate the factors influencing their behaviours. Forty caregivers will be selected stratified by those who adhered to medicines and the day 3 post-treatment review and those who did not. A semi-structured interview guide with open-ended questions will be used to ensure that key topics are covered as well as allow flexibility to explore issues raised by participants during the interview. All interviews will be digitally recorded, with the audio files subsequently downloaded onto a computer and transcribed into Word. The data will be entered in NVivo software for data management and a framework approach will be adopted in the analysis. The process of analysis will involve familiarization with the data, development of initial codes based on the research questions and issues emerging from the data, refinement of codes, and their allocation to the primary and emerging themes. In addition, in-depth interviews will be conducted with eight health workers to get their views about the feasibility and anticipated burdens of the day 3 post treatment reviews in routine settings.

Finally, a cost-effectiveness analysis of the intervention with respect to the primary outcomes will be conducted. Costs will be measured under trial conditions, from the provider’s perspective and will include costs required for the Kenya ministry of health to expand the delivery of the intervention nationally. Cost data will be collected using standard procedures and unit costing menus [32] and will distinguish between intervention delivery costs and development/research/evaluation costs [33]. Intervention delivery costs will include costs to develop the text-message delivery system, market costs of text-messages, as well as the costs of providing the day 3 post treatment review, including costs for health worker time, costs for consumables, delivery and storage costs. These costs will be collected from financial reports and the number of SMS delivery reports and market cost per SMS delivered. Cost-effectiveness will be measured as an incremental cost-effectiveness ratio per additional child adhering to AL that is attributed to the intervention.

## Conclusion

In order to avoid long time lags between the generation of research evidence and its uptake into policy and practice, there is a need to think strategically about the impact of research. There are a few good examples where researchers have closely engaged policy makers to improve evidence-led malaria case management policies in east Africa. In the late 1990s, there was evidence from research demonstrating widespread malaria parasite resistance to monotherapy. However, despite the over whelming evidence, there was no change in treatment policy. In view of the failure of research to impact policy, the East African Network for Monitoring Antimalarial Treatment (EANMAT) was established and generated credible evidence that supported the change in policy from monotherapy to ACT. The success of EANMAT was attributed to bringing together national malaria control programmes and research stakeholders [34,35]. If this research is successful, this EANMAT experience will be used to facilitate the convening of joint fora where researchers and policy makers meet and interact through policy-science dialogues, active dissemination of synthesized information, provision of policy briefs, as well as, indirect communication through appropriate media to ensure that the research findings inform policy and practice.

The uptake of research evidence into policy can further be facilitated if policy makers/implementers are actively engaged from the design of research and agenda setting. This research was designed with the close involvement of the malaria control unit and the devolved districts in Kenya so as to get their buy in. If the trial is successful, the research team will work in partnership with the national malaria control programmes, the roll back malaria (RBM) partnership, and the World Health Organization’s regional and country offices to ensure that the results are translated into policy and practice locally and regionally. Researchers on our team have established close working relationships with the Kenya ministry of health and other ministries of health in the sub-region and will use this experience to facilitate the uptake of our research evidence into policy in Kenya and the region.

Policy makers and researchers by the nature of their motivations have different roles. However the two groups should talk closely to each other and strive for a better mutual understanding. Policy makers/implementers often have wider horizons, are curious about other solution options and may not rely on only one type of research. Moreover, there are other aspects-economic, cultural, behavioral and social, that impact policy uptake. The research described in this protocol is multi-disciplinary and involves investigating different aspects-efficacy, social, cost and cost effectiveness, so it is likely to generate diverse and critical information to enable policy makers to take informed decisions.

Finally, the increasing use of new m-Health technologies to improve healthcare deliver in Africa is commendable. However, the introduction of m-Health technologies into health care delivery should be based on a robust evidence base of their efficacy, cost, effectiveness, feasibility and scalability. The scientific rigor required in assessing their impact, the assumptions that underpin their simplicity, low cost and effectiveness should not be less than would be required for any other new health intervention.

## Figures and Tables

**Table 1:** Timing and content of messages.

Confirmation message (at the time of dose 1 on Day 0)	Hello (CAREGIVER NAME), this message is to confirm that you are now registered into the SMS malaria study. Thank you (HF Name)
Dose 2 reminder (8 hours after dose1 on Day 0)	Hello (CAREGIVER NAME), have you remembered to give your child dose 2 of malaria medicine? If not, please do so. Thank you, (HF Name)
Dose 3 reminder (8.00 am on Day 1)	Hello (CAREGIVER NAME), have you remembered to give your child dose 3 of malaria medicine? If not, please do so. Thank you, (HF Name)
Dose 4 reminder (8.00 pm on Day 1)	Hello (CAREGIVER NAME), have you remembered to give your child dose 4 of malaria medicine? If not, please do so. Thank you, (HF Name)
Dose 5 reminder (8.00 am on Day 2)	Hello (CAREGIVER NAME), have you remembered to give your child dose 5 of malaria medicine? If not, please do so. Thank you, (HF Name)
Dose 6 reminder (8.00 pm on Day 2)	Hello (CAREGIVER NAME), have you remembered to give your child dose 6 of malaria medicine? If not, please do so. Thank you, (HF Name)
Day 3 review reminder^*^ (8.30 pm on Day 2)	Hello (CAREGIVER NAME), please remember to bring the child back to hospital tomorrow to confirm clearance of malaria parasites. Thank you, (HF Name)
Day 3 review reminder^*^ (8.00 am on Day 3)	Hello (CAREGIVER NAME), please remember to bring child back to hospital today to confirm clearance of malaria parasites. Thank you, (HF Name)
Day 7 unscheduled visit reminder (8.00 am on Day 7)	Hello (CAREGIVER NAME), I hope the child is doing well. If not, please bring them back to the hospital as soon as possible. Thank you, (HF Name)
Day 14 unscheduled visit reminder (8.00 am on Day 14)	Hello (CAREGIVER NAME), I hope the child is doing well. If not, please bring them back to the hospital as soon as possible. Thank you, (HF Name)
Day 21 unscheduled visit reminder (8.00 am on Day 21)	Hello (CAREGIVER NAME), I hope the child is doing well. If not, please bring them back to the hospital as soon as possible. Thank you, (HF Name)
Day 28 review reminder (6.00 pm on Day 27)	Hello (CAREGIVER NAME), Please bring your child back to the hospital tomorrow for day 28 review as advised by the doctor. Thank you, (HF Name)
Day 28 review reminder (8.00 am on Day 28)	Hello (CAREGIVER NAME), Please bring your child back to the hospital today for day 28 review as advised by the doctor. Thank you, (HF Name)

* Day 3 review reminders are not sent to the Category 3 patients since they are reviewed at home
